# FAMILY CAREGIVING IN LTC SETTINGS DURING THE PANDEMIC: COMMUNICATION CHALLENGES AND LEVERAGING TECHNOLOGY

**DOI:** 10.1093/geroni/igac059.108

**Published:** 2022-12-20

**Authors:** Gashaye Melaku Tefera, Erin Robinson, Geunhye Park

**Affiliations:** University of Missouri, Columbia, Missouri, United States; University of Missouri, Columbia, Missouri, United States; University of Missouri, Columbia, Missouri, United States

## Abstract

COVID19 related lockdown and protocols caused disruptions in family caregiving for older adults living in LTC settings. However, there is a paucity of research on the challenges and experiences of family caregivers in maintaining their caregiving role during the pandemic. Hence, this qualitative study explores family caregivers’ communication challenges and the role technology played in performing their caregiving roles. One-on-one in-depth interviews (N=25) were conducted with family caregivers (Mean age= 59.7; 92% female; 76% child) via phone/Zoom. Interviews were transcribed and thematically analyzed using Nvivo12. Findings demonstrate that family caregivers of older adults in LTC settings experienced severe communication barriers with staff at those facilities in the early onset of the pandemic, including delays of important information about their care recipients. Participants highlighted high staff turnover, inadequate training, staff unfamiliarity with technology, and poor internet connections as perpetuating communication barriers. During this time, their older care recipients experienced visual and hearing impairments that affected their ability to communicate, as well as cognitive decline. Despite this, family caregivers were able to successfully utilize various forms of technology to continue providing care supports and social support to their loved ones. Although participants relied on phone calls and email communications, they also used other platforms including Zoom, FaceTime, Nixplay, and TextNow. Participants used devices including landline phones, cellphones, computers, tablets, Ipads, and walkie-talkies to execute their communication. Implications of this study suggest that improving access and utilization of technology in LTC settings can enhance family caregiving during unprecedented events like the COVID19 pandemic.

